# 

**Published:** 1846-05-01

**Authors:** 


*To our Readers and Correspondents.*—This is the last number of our first volume. It is befitting that we return our thanks to our readers and patrons who have thus far encouraged and sustained our undertaking. It is still more becoming that we tender our acknowledgements to our friends who have furnished contributions for the work, since we are well aware it is to them that the credit is chiefly due for whatever interest and value the journal may have possessed. To both readers and correspondents, then, we beg leave to express our grateful sense of their favors. Thanks to the good offices of both, we do not feel called upon at this time to make a valedictory. The expiration of our first volume is not the occasion of a final leave-taking, but, on the other hand, as our readers and correspondents are aware, it precedes the commencement of larger enterprise. We trust it is not indecorous to express the hope that they who have accompanied and assisted us thus far, will continue their companionship and co-operation. Our correspondents have pledged their continued efforts: will our readers one and all, do so likewise; not only remaining themselves patrons, but endeavoring to increase the list by their recommendations and influence among their friends? We should assuredly not make this request if our undertaking were a scheme for personal profit. There would, of course, be no impropriety in the fact if it were so. Editorial labors are certainly not less worthy of pecuniary compensation than any other. As it is, however, we deem it due to ourselves to say, that we expect to make a greater pecuniary sacrifice than we ask from our subscribers, assuming that we shall not render an equivalent for what they pay, and in addition to this it will readily be conceived that the amount of labor which we impose on ourselves is not small. We shall, however, welcome the increased duties which are in reserve for us with cheerfulness, if we can have the satisfaction to know that we succeed in performing them acceptably, and that we are sustained in our efforts to make the journal more valuable and useful. We take this occasion to repeat, that whatever surplus of pecuniary avails there may be over the expenses of publication will be devoted to an increase in the size of the journal, or to its improvement in some other mode. We hope our readers will recollect this when they deliberate upon the propriety of continuing the work with its increased subscription price; and, consider that the amount is insignificant when compared with the editorial labors which are gratuitously rendered.

